# Molecular Characterization of Fungal Pigments

**DOI:** 10.3390/jof7050326

**Published:** 2021-04-23

**Authors:** Miriam S. Valenzuela-Gloria, Nagamani Balagurusamy, Mónica L. Chávez-González, Oscar Aguilar, Ayerim Hernández-Almanza, Cristóbal N. Aguilar

**Affiliations:** 1 School of Biological Sciences, Universidad Autónoma de Coahuila, Torreón 27000, Coahuila, Mexico; miriamvalenzuela@uadec.edu.mx (M.S.V.-G.); bnagamani@uadec.edu.mx (N.B.); 2 Bioprocesses and Bioproducts Research Group, BBG-DIA, Food Research Department, School of Chemistry, Universidad Autónoma de Coahuila, Saltillo 25280, Coahuila, Mexico; monicachavez@uadec.edu.mx; 3 Tecnológico de Monterrey, Escuela de Ingeniería y Ciencias, Av. Eugenio Garza Sada 2501 Sur, Monterrey 64849, Nuevo León, Mexico; alex.aguilar@tec.mx

**Keywords:** fungi pigments, types, structure, molecular elucidation

## Abstract

The industrial application of pigments of biological origin has been gaining strength over time, which is mainly explained by the increased interest of the consumer for products with few synthetic additives. So, the search for biomolecules from natural origin has challenged food scientists and technologists to identify, develop efficient and less consuming strategies for extraction and characterization of biopigments. In this task, elucidation of molecular structure has become a fundamental requirement, since it is necessary to comply with compound regulatory submissions of industrial sectors such as food, pharmaceutical agrichemicals, and other new chemical entity registrations. Molecular elucidation consists of establishing the chemical structure of a molecule, which allows us to understand the interaction between the natural additive (colorant, flavor, antioxidant, etc) and its use (interaction with the rest of the mixture of compounds). Elucidation of molecular characteristics can be achieved through several techniques, the most common being infrared spectroscopy (IR), spectroscopy or ultraviolet-visible spectrophotometry (UV-VIS), nuclear-resonance spectroscopy (MAGNETIC MRI), and mass spectrometry. This review provides the details that aid for the molecular elucidation of pigments of fungal origin, for a viable and innocuous application of these biopigments by various industries.

## 1. Introduction

At present, the colorants are employed by various industrial sectors such as textiles, pharmaceuticals, nutraceuticals, and most importantly as an additive in the food industry. The use of colorants by food products contributes widely to the visual impact to gain the attention and preference of the consumer, apart from adding value to the product. [1] The use of these agents has a number of disadvantages, including a lack of raw materials, differences in pigment extraction, and, most notably, the environmental effect of chemical syntheses used in the production of these additives. Recently, research has been focusing on the quest for new natural sources, and which has thrown up a myriad of potential sources such as plants, animals, bacteria, microalgae and even fungi.

Pigments derived from microbes have many benefits over those derived from plants or animals, including low environmental effects, viability, profitability, and ease of handling prior to, during, and after processing [2]. Fungi stand out among microorganisms of interest because of their ability to produce a broad spectrum of soluble pigments under a variety of conditions and substrates [3]. Because of their ease of cultivation in the laboratory and comparatively lean downstream operations that are readily scalable at pilot or plant scales, pigments extracted from filamentous fungi have piqued industrial interest [4].

Fungal pigments have the capacity to be a significant source of biopigments due to their high yield potential and ease of extraction. For example, the biomass production of *Chlamydomonas reinhardtii* microalgae oscillates in the range of 2.0 g L^−1^ in dry biomass [5], whereas the biomass production of a filamentous fungus such as *Mucor circinelloides* oscillates in a radius of 4.0 g L^−1^ in dry biomass [6]. About the fact that all previous values are in crops without optimization assessment, it is possible to find a 1:2 relationship in the production of dry biomass. According to Zhang et al. [7], *Monascus* will increase its yields from 48.4 to 215.4 mg L^−1^ by optimizing the glutamic acid present in the culture medium, which is equal to a 1:3.5 ratio [7]. As a result, understanding and characterizing the molecular structure of these pigments for safe and sustainable use becomes critical if mass application is intended [2].

In general, the qualitative nature of the pigments are studied using comparative charts, or colorimeter or by use of spectrophotometry. However, qualitative color assessment only helps one to make assumptions; therefore, understanding and investigation of pigments at the molecular level are needed for later use.

The present review provides an overview of fungal pigment production as well as an analysis of the current analytical methodologies commonly used for the chemical and structural characterization of these new pigments and other additives in order to gain permission from the regulatory agencies in charge of regulating their use as additives in the agro-food-pharmaceutical industries.

## 2. Fungi as Biopigment Producers

Fungi are one of the kingdoms of the Eukarya domain that can be found in almost any climate, especially in terrestrial ecosystems [8]. They play an important role in the nitrogen cycle because they are scavengers, decomposers, predators, pathogens, and even parasites, and they can survive in symbiotic relationships with plants, algae, and animals, among others. Some fungi genera have grown in importance over time due to their ability for industrial applications [8].

Fungi, especially filamentous fungi, have gained popularity due to their ability to produce a diverse range of secondary metabolites that are important in the health, food, agricultural, and other sectors. Biopigments are one of them, and they are being studied because of their biodegradable nature, low production costs, wide range of colors, and biological properties ranging from antioxidants to anticancer [6,8]. Most fungi produce water-soluble pigments that are suitable for industrial production since they are easy to scale-up in industrial fermenters and can be extracted without the use of organic solvents [9].

Some of the most important fungal species or genera? for pigment production are found in the families, they are as follow: *Monascaceae, Trichocomaceae, Nectriaceae, Hypocreaceae, Pleosporaceae, Cordycipitaceae, Xylariaceae, Chaetomiaceae, Sordariaceae, Chlorociboriaceae, Hyaloscyphaceae, Hymenochaetaceae, Polyporaceae, Ophiostomataceae, Tremellaceae, Neurosporaceae*, and *Tuberaceae* [7,9,10,11]. These metabolites are generated by *Monascus* spp. in general through the polyketide pathway, which is directly linked to fatty acid biosynthesis. Though, *Neurospora* spp. does so through the carotenoids’ biosynthetic pathway. *Monascus* spp. is commonly used as a model fungus for the assessment of its biosynthetic pathway at the pilot level; but, owing to its difficulty, it has not yet been completely elucidated. On the other hand, some species, such as *Fusarium* spp., can produce pigments through the polyketide and carotenoid biosynthetic pathways [4,7,9]. Table 1 shows several fungal species and their pigment production. 

## 3. The Most Common Fungal Pigments and Their Properties

Obtaining pigments from natural sources is an activity that has been done for a long time, and in recent years has proven to be a great solution to avoiding the environmental impact caused by the production of synthetic colorants. Some of the most widely isolated and used natural pigments are carotenoids, anthocyanins, chlorophylls, phycobiliproteins, betalains, and also quinones. The natural origins of these pigments are diverse; however, microorganisms emerge due to their ease of cultivation and extraction, as well as their large genetic diversity [23].

Because of their high pigment production yields, fungi have managed to gain a notable position within the diverse range of microorganisms pigment producers. Which fungi produce in the form of secondary metabolites under various stress conditions. Fungal pigments can be categorized as carotenoids or polyketides depending on their chemical composition. The fungal polyketides are made up of tetraetides and octaetides, which form eight C_2_ units to form the polyketide chain, while carotenoids are made up of terpenoids, which comprise forty carbons in their main chain [10,24].

Pigments derived from fungal metabolism not only have dyeing properties, but they also have a number of beneficial properties that enhance their effects, such as anti-oxidant and antitumor activity to name a few examples [25]. *Aspergillus, Fusarium*, *Penicillum, Trichoderma*, and *Monascus* are some of the most widespread pigment-producing fungal genera. Some fungi can produce a variety of pigments depending on their growth conditions. To put it another way, the synthesis of pigments by fungi is critical to their growth since the production of these secondary metabolites is primarily a photoprotection mechanism by the microorganism. Different colorimetric ranges are obtained depending on the genus and/or species, which end up being important descriptive characteristics of each one. For example, the green color of *Penicillium*, the violet color of *Cortinarius*, the yellow, orange, and red color of *Monascus* are its distinguishing features. Many fungal pigments are quinones or similar conjugated structures [7,13]. Quinones are produced by fungi through the polyketide pathway. Fugigatin from *Aspergillus fumigatus* is another example, which is a polyketide found in Nature. The latter can also synthesize two other pigments from the same polyketide route; however, this occurs under different stress conditions; both pigments are members of the hydroquinone family (auroglaucin and flavoglaucin) [26]. Another clear example is the synthesis of pigments by *Monascus*, which has the ability to synthesize six distinct polyketide pigments with colors ranging from yellow to red; where monascin and ankaflavin have an amber color, while monascorubrin and rubropunctatin have an orange color, and finally, monascorubramine and rubropuntamine is become a reddish color [9,15,16].

## 4. Problems of Mycotoxin Production in Pigment Producing Fungi

The production of pigments by filamentous fungi has piqued the attention of industry, not only as a value-added commodity for biorefineries, but also as an alternative to synthetic pigments due to the growing demand for natural pigments, especially by the food industry [1].

**Figure 1:**
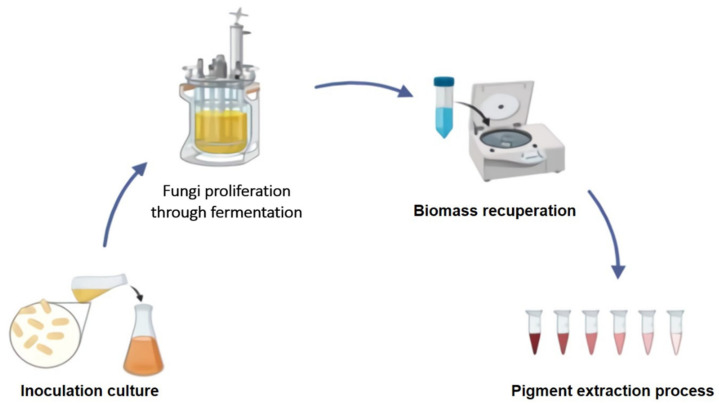
A general scheme for the production of fungal pigments under established controlled conditions.

One of the benefits of microbial pigments is that they are simple to produce. Once the strains are selected, optimal growth conditions for shorter generation period and high yields can be achieved, as can the use of agro-industrial residues. The optimization of the production and subsequent processes is strain specific [7,9]. Several experiments have concentrated on the structure of the growth medium and difference in culture conditions to increase the yield of these compounds [7,17,24], while others have focused on taxonomic identification of fungi and chemical characterization of products before choosing these strains for industrial commercialization [27]. Figure 1 depicts a general short scheme for the production of fungal pigments under established regulated conditions.

The toxicity of the compounds, which occurs when the pigment is bound to a collection of interferents known as impurities, is a significant disadvantage that can impede processing or the establishment of a commercial process. Some strains not only contain useful pigments, but also a number of mycotoxins [12]. In general, safety regulatory authorities play a key role in overseeing the use of such strains based on previous cytotoxicity studies [11]. Several species of *Penicillium, Eurotium, Fusarium* are among the microorganisms that can produce toxic metabolites [28]. As a result, the possible production of mycotoxins is a significant issue that restricts the commercial use of these fungal strains. This problem, along with the increasing demand for natural colorant alternatives from both consumers and regulators, has prompted research and analysis of other genera of potential pigment-producing fungi. Thus, in order to expand the applications of pigments produced by fungi, researchers have focused on edible fungi that can naturally synthesize and secrete pigments with a lower propensity for toxic compound synthesis.

## 5. Downstream Processing of Fungal Biopigments

The next step is the isolation and recovery of produced pigments after the pigments are produced by various microorganisms. Through time, an infinite number of chemical solvents have been used to remove pigments and other forms of bioactive compounds. However, today’s consumer expects a product free of chemical residues due to the health risks and environmental pollution that its toxicity can cause. Traditional extraction techniques necessitate the use of high temperatures and long exposure periods, which affects the stability of molecules [13,14]. Different methods for the recovery of metabolites of concern have been explored as a result of these new toxicity-free criteria and some international regulations. The use of a green solvent means shorter operation periods and less waste production. Carotenoids are among the pigments most widely produced by fungi. Table 2 shows some new and more environmentally sustainable carotenoid extraction processes.

Although the extraction process is not the focal point of the research in fungal pigments, it has earned the title of critical point and if it is not carried out properly and safely, it would be very difficult to proceed with the study at the same time. Molecular elucidation step, since the greatest possible precision in sample preparation is needed in this phase to obtain a stable and effective molecular elucidation.

## 6. Molecular Elucidation of Fungal Pigments

### Importance of Molecular Elucidation

Information of a compound’s chemical and molecular structure is critical because it describes the conformation of a chemical structure and allows for the establishment of the forms of relations that regulate the structures. Furthermore, it enables one to comprehend how certain atoms and bonds combine to form various functional groups. All of this knowledge enables one to understand and model the physical and chemical properties of chemical structures. This knowledge, if obtained in a timely manner, would allow for the description of the pigments’ stability behavior as well as the quest for better matrices to contain them, thus extending their useful life.

One of the shortcomings of the chemical characterization of pigments of microbial origins is the lack of industry reference criteria that enable adequate characterization of the compounds formed by various microorganisms. This is a field of possibility for businesses and/or laboratories interested in producing microbial pigments, as well as a critical challenge that must be completed before reaching a commercial level.

On the other hand, there are various analytical instruments available today to determine the chemical structure of compounds. These methods allow us to determine the molecular weights of molecules, the types of bonds that engage in molecular conformation, the polarity of the structures, and their functional groups etc. The selection of the approach and/or set of analytical methodologies is an essential step in the proper description of the structures. The most important analytical methodologies for elucidating the chemical structures of microbial pigments are described below.

## 7. Methodologies for Elucidation of Chemical Structure of Pigments

### 7.1. Spectrophotometric Methods

According to the Royal Spanish Academy, spectrophotometry is a scientific method widely used to determine how much light a chemical compound absorbs and is based on the Beer-Lambert law. The Beer-lambert law is a combination of the other four laws (Bouguer, Bunsen, Roscoe, and Beer) that enabled it to be enunciated. According to Hardesty and Attili [37], “*the intensity of a monochromatic light beam incident perpendicular on a sample decreases exponentially with sample concentration*”. This law states that:

(1) A=K∗C

where, *A* = Sample absorbance; *K*= Constant of wavelength, which is fixed according to the nature of the substance analyzed and the material of the cell used; and *C* = Sample concentration.

Then, if we look at this equation closely, we will see a similarity with the equation of the line, which, since it lacks an interjection point with the coordinates (n), we can infer that it would pass through the origin of the coordinates (standard); where “*K*” is the slope of the line [38].

There are several modifications and adaptations to this process, and we will discuss some of the more widely used spectrophotometric methods for the analysis of fungal pigments below.

#### 7.1.1. Ultraviolet Visible (UV-VIS)

The technique of ultraviolet-visible absorption spectrophotometry (UV-VIS) is based on the attenuation of electromagnetic radiation measurement by an absorbing substance [39]. This radiation has a spectral range of approximately 190-800 nm and varies from other similar regions in terms of energy levels and type of excitation [26,40].The wavelengths that can be detected using this method are classified as UVC, UVB, UVA, and visible; where the UVC wavelength is called short since it covers a range from 100 to 280 nm, UVB is known as the medium wavelength that covers 280 to 315 nm, UVA is known as the very long wavelength that covers 315 to 400 nm, and finally, the visible spectrum can be said to cover from 315 to 400 nm. Furthermore, of course should not forgetting to mention infrared light [38]. Reflection, absorption, and even interference both contribute to this attenuation. However, accurate calculations of this can be made using absorbance databases. In certain ways, the absorbance can be said to be equal to the concentration of the analyte to be measured and to the wavelength of the light as it passes through the sample during irradiation, as regulated by Beer’s Law [25,40]. Since this is a linear relationship, it can be affected by a variety of factors, including the spectrophotometer’s characteristics, photodegradation of molecules, the presence of dispersion or absorption interferences in the sample, fluorescent compounds in the sample, reactions between the analyte and the solvent, and the pH [25,26]. The short wavelength limit is caused by atmospheric gas absorption at ultraviolet wavelengths shorter than 180 nm. When a spectrometer is purged with nitrogen gas, this limit is raised to 175 nm. Working above 175 nm necessitates the use of a vacuum spectrometer and an ultraviolet light source [39].

UV-Vis is one of the most ubiquitous characterization and analytical techniques in science. Its use in materials research could be classified into two categories: (1) quantitative measurements of an analyte in the gas, liquid, or solid phase and (2) characterization of the optical and electronic properties of a material [39]. The first application for quantitative measurements derives from the linear relationship between absorbance and absorbent concentration. Which turns out to be easy and fast since the main requirement is only measuring absorbance or reflectance in a single wavelength. Since most molecular and solid materials have broad absorption characteristics, quantitative measurements are less sensitive to instrumental variables than other analytical methods [26,41]. The identification of an analyte, on the other hand, can be accomplished by matching the absorption spectrum of the unknown substance with graphs or tables of the spectra of known substances. Two or three analytes can be recognized in some cases [42].

Because of the above, this type of spectrophotometry can be used to characterize the absorption, transmission, and reflectance of a wide range of technologically significant materials, such as pigments. The substance in this case is a transparent host with intentional dopants or unintentional impurities that regulate optical absorption [26,40]. Molnàr et al. [43] described the chromophores present in the carotenoids isolated from *Sarcoscypha coccinea* and obtained a characteristic confirmation of such agents including 30, 40 -didehydro-10,20-dihydro-β, ψ-carotene, 30,40-didehydro-10, 20-dihydro-β, ψ-carotene-20-one [43].

#### 7.1.2. Infrared (IR)

Infrared spectrophotometry is the most widely used technique for pilot-level applications. This is done with the aid of an infrared spectrophotometer, which can distinguish molecular structures by producing wavelengths with a spectral range of 500 to 4000 nm. That is, the transmittance response for a given wavelength shows the difference in the links caused by photodegradation [38].

This method works by exposing a sample to ultraviolet radiation, which causes changes in the vibrational states of the sample’s constituent molecules. Radiation absorption by a sample indicates the type of bonds and functional groups present [25,44]. It is useful to divide the infrared region into three regions called near infrared (NIR), middle infrared (MIR), and far infrared (FIR) from the standpoint of instrumentation and applications [44,45]. The vast majority of traditional analytical applications of infrared spectroscopy are focused on the use of the middle infrared (4000-600 cm^−1^) and near infrared, allowing this method to be converted into a quantitative technique. The Fourier transform technique, which converts a time domain spectrum to a frequency domain spectrum using a mathematical operation, allows for the generation of spectra that are quick, accurate, and have elevated signal/noise (S/N) relationships [46].

There are several measurement techniques to obtain this type of spectra; however, some of the most common are described below:

Transmission: IR radiation is passed into the sample in this measurement process, recording the amount of energy absorbed by the sample. The IR spectrum is obtained using a reference experiment by comparing the radiation recorded after going through the sample. With the proper accessories, this method analyzes gaseous, liquid, and solid samples [47].

Reflection: Infrared radiation is reflected on the sample. The sample’s molecular information is extracted by analyzing the reflected radiation and comparing it to the incident radiation. To use this measuring tool, the sample must be reflective or mounted on a reflective surface [44,45].

ATR mode: It is a sampling mode in which an infrared beam is projected onto a crystal with a high refractive index. The beam reflects off the inside of the glass, generating an evanescent wave that enters the sample. This must be in near proximity to the crystal. A portion of the evanescent wave’s energy is absorbed, and the reflected radiation (containing chemical information about the sample) is directed to the detector. It is a very versatile method for measuring liquid and solid samples without manually processing them [47].

In order to gain as much detail as possible on the pigment in question, the methods previously described are usually used as a supplement. As in the case of Quijano-Ortega et al. [48], both FTIR and ATR were used jointly to determine carotenoids present in *Cucurbita* spp., where structural conformations typical of carotenoids could be determined, for example the double bonding between carbons, deformation of CH_3_ groups and even the stable existence of CH_2_ chains [48].

#### 7.1.3. Combined Diffusion (Raman)

Raman spectroscopy is a high-resolution photonic technique that offers chemical and structural knowledge on virtually every organic or inorganic material or compound in a couple of seconds, enabling it to be identified. As a result, the analysis of this method is dependent on the evaluation of the light scattered by a material when a monochromatic beam of light falls on it [49]. This is because a particular portion of light is inelastically dispersed, experiencing minor frequency shifts that are typical of the substance studied and regardless of the frequency of the incident light. It is an inspection procedure that is conducted directly on the sample to be examined without the need for any additional planning and without having any impact on the analyte surface [30,50].

Due to the simplicity of execution of this technique, it is used in many fields of application, if not all, in response to its foundation on molecular vibrations, which take place in any body. Among its multiple fields of application, pigments are positioned in an important place, as organic compounds to determine the conformational macrocomponents of these agents [51]. Nokkaew et al. [52] used the Raman technique to structurally identify the carotenoids present in crude palm oil through the identification of carbon-carbon double bonds (C = C) and their positions in the terpenoid chain [52].

#### 7.1.4. Mass Spectrometry (MS)

Mass spectrometry is a microanalytical method used to identify unknown substances, quantify known compounds, and elucidate molecule structure and chemical properties. It uses small volumes of sample to collect details such as the weight and, in some cases, the structure of the analyte [53]. As a result, the sample is ionized (and thereby destroyed) using different protocols. The electronic impact method is one of the most widely employed, as it involves bombarding the sample (which has traditionally been vaporized using a high vacuum and a heat source) with a high-speed current of electrons, and this is how the substance loses several electrons and fragments, producing various ions, radicals, and neutral molecules [31,32]. The ions (charged molecules or fragments) are then operated by an ion accelerator to a curved analyzer tube with a high magnetic field and then to a collector/analyzer where the impacts of those ions are collected as a function of their *m/z* ratio. Each compound is unique, because each compound can ionize and fragment in a different manner, and mass spectrometry uses this idea to classify each analyte [54,55].

We can use mass spectrometry to determine the chemical composition of samples, the composition of inorganic, organic, and biological compounds, the qualitative and quantitative composition of complex mixtures, the structure and composition of solid surfaces, and even about the isotopic ratios of atoms in the samples [32,34]. Chen et al. [56] employed LC-MS method to confirm the presence of flavonoids on three different types of pigmented cotton fibers.

Among the analytical techniques often used in mass spectrometry, chromatographic methods such as gas chromatography and liquid chromatography coupled to mass spectrometers and isotopic ratio mass spectrometry for the study of stable isotopes deserve special mention (C, N, H, O and S) [31,32,34]. 

The above is used to determine the molecular mass of peptides and proteins, the biomolecular elucidation of structures derived from natural agents such as proteins and also pigments, and the recognition of pairs of proteins that interact using affinity purified mass spectrometry. Proteins are an essential component of biological systems and studying proteins in depth is critical to understanding life and its processes [53].

### 7.2. Magnetic Spectrometry Methods

#### 7.2.1. Nuclear Magnetic Resonance (NMR)

Nuclear Magnetic Resonance (NMR) is one of the most often used magnetic techniques for studying pigments of fungal origin. Whose basis is founded on the assumption that all nuclei with an odd number of protons and neutrons have a magnetic moment and inherent angular momentum or have a spin greater than zero [57]. As a result, this approach is primarily used to extract physical, chemical, electronic, and structural details on molecules.

By applying NMR on fungal pigments, it is possible to know different conformational aspects, such as the following [35,37]:

- To detect the number of protons, present in the pigment molecule (signal number).
- To identify the type of bond that these protons form, whether they are alkanes, alkenes, hydroxyl, aromatic, among others (signal position).
- Allows setting the number of protons generated in each peak/signal (signal intensity)
- To perform signal splitting, either in doublets, triplets and/or multiplets, which will allow in knowing the number of protons attached to the carbon that is next to the transporting carbon of the previously broken signal proton.

The use of this technique has helped over the years to explain some of the many structural details that pigments provide, especially those produced by fungi and some plants. NMR is called a low invasive procedure, which means that the effect it has on the molecular structure of pigments is comparatively low, allowing it to rule out the possibility that they are affected by energetic influences [58,59]. It is widely used in the study of the stability and reactivity of conformational isomers, which would be almost impossible to conduct without its inclusion. The proton (_1_H), _13_C, and _15_N are among the most common nuclei for its realization [35,38,60].

#### 7.2.2. Electron Spin Resonance (ESR)

Electron spin resonance (ESR) is routinely characterized as the amount of radiation absorbed by a static magnetic field; it is often referred to as “paramagnetic resonance” or “paramagnetic electron resonance” [61,62]. Radicals are a special class of paramagnetic molecule. ESR spectroscopy can be used on these. As a result of the ESR technique’s existence, it is possible to apply it to the analysis of free radicals found in pigments, whether or not they are of fungal origin. Free radicals are well-known molecular species that carry an unpaired electron in the paramagnetic group of the atomic orbital region of molecular species. As a result of the above, these are unstable, reactive, and can also cause redox reactions with other molecules [42,63,64]. 

**Figure 2:**
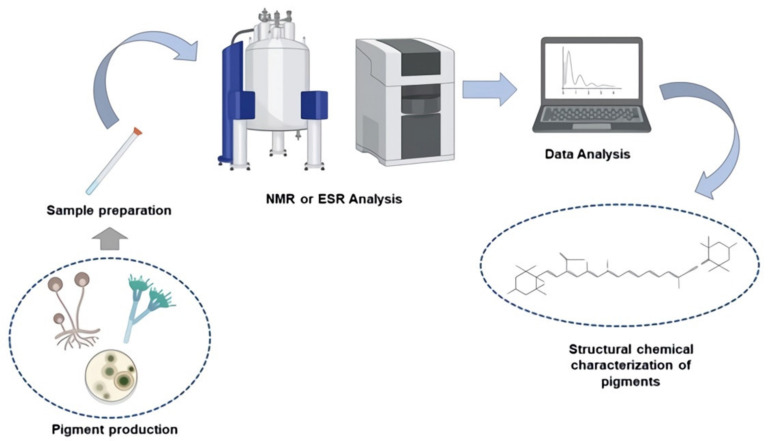
A general diagram of the fungal pigment analysis protocol using NMR and ESR.

Because of its ability to detect free radical absorption, it has been used over time to assess oxidative stability, copper chelating capability, and even radiation damage to biological bodies when exposed to new technologies such as high pressures, electrical pulses, ultrasound, and so on [53,65,66]. According to Gonçalves et al., the application of ESR in the melanins formed by *Aspergillus nidulans* is stated, with G values (2,007) identifying that the stability and vibrations produced by said pigments are linked to the presence of C=C and C=O groups [67]. In Figure 2, we can see a brief diagram of how the methods discussed in this section are implemented.

### 7.3. Chromatographic Methods

#### 7.3.1. High Performance Liquid (HPLC)

The chromatography principle essentially governs the separation of the components of a mixture; this from a mobile phase fluid (gas, liquid, or supercritical fluid), which drags the sample at constant flow and pressure due to the use of a pump. This pressure flow leads to a column containing the stationary phase, which is either a solid or a liquid bound to a solid [68]. As a result of the interaction between the components of the mixture and the two phases, they allow separation; hence, at the end, it passes through a detector that produces a signal that allows both indicating the moment of appearance of the various components that make up the sample and qualifying it quantitatively and qualitatively [69].

HPLC differs from other forms of chromatography in that it is not limited by the sample’s volatility or thermal stability. The main explanation for this is that it is broadly applicable for the study of macromolecules, such as fungal pigments in this case [43,70]. Several authors have reported the use of this method in the molecular characterization of fungal pigments at various stages. Beginning with Lebeau et al. [21], who used UV-VIS and HPLC-DAD (inverse) to identify and elucidate the conformation of two new pigments from *Fusarium oxysporum*, which they called wild-type purple naphthoquinone and bikaverin, respectively [21]. Venkatachalam et al. [22], reported the application of HPLC in some of its variants (PDA-ESI/MS) for the analysis of the pigments produced by *Talaromyces albobiverticillius* 30548 and reported twelve compounds of *Monascus* spp. Further, they characterized and elucidated a new compound called NGABA-PP-V (6-[(Z)-2-Carboxyvinyl] -N-GABA-PP-V) with a *cis* configuration at the C10-C11 carbon double bond [22]. Finally, Gonçalves et al. [71] contributed by identifying and quantifying the phenolic compounds gallic acid, catechin, chlorogenic acid, caffeic acid, and vanillin found in the ethyl extract of *Penicillium flavigenum* CML2965 using HPLC [71]. Since it is a non-limiting method, it is obvious that there are millions of works published in its use for the molecular elucidation of fungal pigments, which is why the examples above are just a few of the most recent.

#### 7.3.2. Thin Layer (TLC)

Thin layer chromatography (TLC) is one of the oldest and most commonly used methods in the molecular analysis of biopigments, including those derived from fungi. This procedure involves chromatography in a stationary phase in the form of a thin layer, usually on aluminum, glass, and also plastic surfaces, where a standard solution is added and expressed as a narrow band on the thin layer of adsorbent as an indicator that it has been distributed evenly on the support/surface. This technique consists of chromatography carried out in a stationary phase in the form of a thin layer, commonly on aluminum, glass, and even plastic surfaces and with solvents as mobile phase. The separated molecules are detected after the evaporation of the solvent and by employing physical methods or chemical staining reagents [46,48,72,73].

Thin layer chromatography allows for the manipulation of extracts of different types, whether crude or pure; additionally, the equipment used is simple, inexpensive, and effective. And also, this method can be used quantitatively, where the components are isolated on the TLC tray, to be subsequently removed with an appropriate solvent, such as ethanol or acetone, and then lead to the application of a procedure using a chromogen, and thus be measured by spectrophotometry at a determined wavelength [74,75]. There are other ways for TLC quantification and detection, such as densitometry under the appropriate conditions, which is commonly used for the study of pigments, mostly anthocyanins and vitamin A precursors [76]. Another simple example is its application to the complete isolation of pigments, most notably those derived from organisms such as *Monascus*, which do not contain only one kind of pigment [77]. Finally, TLC has been used in conjunction with other approaches to increase the belief in carotenoid identifications. For example, Wang et al. [13] used both column chromatography and TLC to investigate the pigments of aeciospores of *Cronartium fusiforme* fungi [13].

## 8. Future Trends and Challenges

Certainly, the market for chemical-free pigments has increased significantly in industry and science. As a result, the extraction from biological origin has gained scientific interest, resulting in alternative processing and extraction from different strains of fungi. Since these have a number of significant advantages, such as low processing costs, high yields, and strong adaptability to shifting substrates, the exploitation of agro-industrial waste has emerged. This is how fungi are able to be considered sustainable industries for the production of pigments, where substantial progress has been made in the optimization of these processes by study and the implementation of different experiments. It is well known that different species of fungi collected from natural sources have a diverse chromatographic spectrum of pigments that are closely linked to a number of biological activities of importance, such as the antioxidant activity present in some pigments. Despite the fact that the area of optimizing processes that produce fungal pigments is progressing at a rapid pace. Another collection of investigations and projects is devoted solely to the structural, magnetic, and interactional information of them, i.e., their classification at the molecular level of fungal pigments, in which a variety of techniques are used, either individually or collectively. To introduce these to a large scale, they must be not only commercially feasible, but also safe, harmless, and controlled.

As a result, society is becoming more mindful of the environmental and health harm that chemically synthesized pigments can cause. The manufacture of fungal pigments has taken a big step forward. However, it is already safe to state that said development is in progress but, due to production conditions, it has not been possible to completely cover existing consumer demand. Thus, achieving total availability by other means is one of the industry’s biggest obstacles. For example, the discovery and investigation of other genera and/or species of fungi that have been underutilized due to ignorance of the existence of their secondary metabolites, which can be elucidated by either of the previously described techniques thus ensuring optimal process optimization.

## Figures and Tables

**Table 1:** More commonly produced pigments and their fungal species.

Pigment Produced		Fungal Species	Reference
**Carotenoids**	β-carotene	*Blakeslea trispora*	[10,12,13]
		*Rhodosporidium* spp.	
		*Rhodotorula* spp.	
		*Penicillium* spp.	
		*Aspergillus giganteus*	
		*Sclerotium* spp.	
		*Sporodionolus pararoseus*	
	Astaxanthin	*Phaffia rhodozyma*	[14,15]
		*Haematococcus pluvalis*	
		*Agrobacterium aurantiacum*	
	Lycopene	*Blakeslea* spp.	[9,13,15,16]
		*Rhodotorula* spp.	
		*Mucorales* spp.	
		*Phycomyces* spp.	
**Polyketides**	Anthraquinones	*Aspergillus* spp.	[9,17,18]
		*Eurotium* spp.	
		*Emericella* spp.	
		*Fusarium* spp.	
		*Penicillium* spp.	
		*Mycospharella* spp.	
		*Microsporum* spp.	
	Hydroxyanthraquinone	*Aspergillus* spp.	[9,17,19]
		*Microsphaeropsis* spp.	
		*Geosmithia* spp.	
		*Trichoderma* spp.	
		*Verticicladiella* spp.	
		*Guignardia* spp.	
	Naphthoquinones	*Chlorociboria* spp.	[9,20,21]
		*Monascus* spp.	
		*Trichoderma* spp.	
		*Fusarium* spp.	
	Azaphilones	*Monascus* spp.	[9,22]
		*Penicillium* spp.	
		*Talaromyces* spp.	
		*Chaetomium* spp.	

**Table 2:** Some recent methods for the extraction of carotenoids.

Microorganism	Extraction Method	Carotenoid Produced	Reference
*Rhodotorula toruloides* NRRL Y-1091	Saponification with alcoholic KOH and hexane	Carotenoids	[29]
*R. glutinis*	Supercritical CO_2_	β-carotene and torularhodin esters	[30]
*R. glutinis* ATCC 2527	Manosonication	Total carotenoids	[31]
*R. glutinis* ATCC 2527	Pulsed electric field	Total carotenoids	[32]
*R. glutinis* P4M422	Bead mill	Lycopene	[33]
*Sporobolomyces ruberriums* H110	French pressure cell	Torularhodin, torulene, β-carotene, γ-carotene	[34]
*Blakeslea trispora*	Steam-explosion-assisted	Total carotenoids	[35]
*Phaffia rhodozyma* NRRL Y-17268	Diatomaceous earth and enzymatic lysis	Total carotenoids	[36]

## References

[1] Mapari S.A., Nielsen K.F., Larsen O.T., Frisvad J.C., Meyer A.S., Thrane U. (2005). Exploring fungal biodiversity for the production of water-soluble pigments as potential natural food colorants. Curr. Opin. Biotechnol..

[2] Kumar A.H., Shankar Vishwakarma J., Singh S.D., Kumar M. (2015). Microbial pigments: Production and their appli-cations in various industries. Int. J. Pharm. Chem. Biol. Sci..

[3] Abdulkadir N., Usman N.A.H.M., Gani H.M.M.M. (2017). Bacterial Pigments and its Significance. MOJ Bioequiv. Bioavailab..

[4] Souza P.N.D.C., Grigoletto T.L.B., De Moraes L.A.B., Abreu L.M., Guimarães L.H.S., Santos C., Galvão L.R., Cardoso P.G. (2016). Production and chemical characterization of pigments in filamentous fungi. Microbiology.

[5] Kong Q.-X., Li L., Martinez B., Chen P., Ruan R. (2009). Culture of Microalgae Chlamydomonas reinhardtii in Wastewater for Biomass Feedstock Production. Appl. Biochem. Biotechnol..

[6] Vicente G., Bautista L.F., Rodríguez R., Gutiérrez F.J., Sádaba I., Ruiz-Vázquez R.M., Torres-Martínez S., Garre V. (2009). Biodiesel production from biomass of an oleaginous fungus. Biochem. Eng. J..

[7] Zhang C., Liang J., Yang L., Chai S., Zhang C., Sun B., Wang C. (2017). Glutamic acid promotes monacolin K production and monacolin K biosynthetic gene cluster expression in Monascus. AMB Express.

[8] Naranjo-Ortiz M.A., Gabaldón T. (2019). Fungal evolution: Major ecological adaptations and evolutionary transitions. Biol. Rev..

[9] Venil C.K., Velmurugan P., Dufossé L., Devi P.R., Ravi A.V. (2020). Fungal Pigments: Potential Coloring Compounds for Wide Ranging Applications in Textile Dyeing. J. Fungi.

[10] Arikan E.B., Canli O., Caro Y., Dufossé L., Dizge N. (2020). Production of bio-based pigments from food processing industry by-products (apple, pomegranate, black carrot, red beet pulps) using *Aspergillus carbonarius*. J. Fungi..

[11] Fox E.M., Howlett B.J. (2008). Secondary metabolism: Regulation and role in fungal biology. Curr. Opin. Microbiol..

[12] Gmoser R., Ferreira J.A., Lennartsson P.R., Taherzadeh M.J. (2017). Filamentous ascomycetes fungi as a source of natural pigments. Fungal Biol. Biotechnol..

[13] Wang E., Dong C., Park R.F., Roberts T.H. (2018). Carotenoid pigments in rust fungi: Extraction, separation, quantification and characterisation. Fungal Biol. Rev..

[14] Gong M., Bassi A. (2016). Carotenoids from microalgae: A review of recent developments. Biotechnol. Adv..

[15] Avalos J., Limón M.C. (2015). Biological roles of fungal carotenoids. Curr. Genet..

[16] Feofilova E.P., Tereshina V.M., Memorskaya A.S., Dul’Kin L.M., Goncharov N.G. (2006). Fungal lycopene: The biotechnology of its production and prospects for its application in medicine. Microbiology.

[17] Lagashetti A.C., Dufossé L., Singh S.K., Singh P.N. (2019). Fungal Pigments and Their Prospects in Different Industries. Microorganisms.

[18] Velíšek J., Cejpek K. (2011). Pigments of higher fungi—A review. Czech J. Food Sci..

[19] Adeboye P.T., Bettiga M., Olsson L. (2014). The chemical nature of phenolic compounds determines their toxicity and induces distinct physiological responses in Saccharomyces cerevisiae in lignocellulose hydrolysates. AMB Express.

[20] Mukherjee G., Mishra T., Deshmukh S.K. (2017). Fungal Pigments: An Overview. Developments in Fungal Biology and Applied Mycology.

[21] Lebeau J., Petit T., Clerc P., Dufossé L., Caro Y. (2019). Isolation of two novel purple naphthoquinone pigments concomitant with the bioactive red bikaverin and derivates thereof produced by Fusarium oxysporum. Biotechnol. Prog..

[22] Venkatachalam M., Zelena M., Cacciola F., Ceslova L., Girard-Valenciennes E., Clerc P., Dugo P., Mondello L., Fouillaud M., Rotondo A. (2018). Partial characterization of the pigments produced by the marine-derived fungus Talaromyces albobiverticillius 30548. Towards a new fungal red colorant for the food industry. J. Food Compos. Anal..

[23] Zhou Z.-Y., Liu J.-K. (2010). Pigments of fungi (macromycetes). Nat. Prod. Rep..

[24] Gmoser R., Ferreira J.A., Taherzadeh M.J., Lennartsson P.R. (2019). Post-treatment of fungal biomass to enhance pigment production. Appl. Biochem. Biotechnol..

[25] Goodwin T.W. (1952). Fungal carotenoids. Bot. Rev..

[26] Latge J.-P., Chamilos G. (2020). Aspergillus fumigatus and Aspergillosis in 2019. Clin. Microbiol. Rev..

[27] Mapari S.A., Meyer A.S., Thrane U., Frisvad J.C. (2009). Identification of potentially safe promising fungal cell factories for the production of polyketide natural food colorants using chemotaxonomic rationale. Microb. Cell Factories.

[28] Heo Y.M., Kim K., Kwon S.L., Na J., Lee H., Jang S., Kim C.H., Jung J., Kim J.-J. (2018). Investigation of Filamentous Fungi Producing Safe, Functional Water-Soluble Pigments. Mycobiology.

[29] Liu Z., Berg C.V.D., Weusthuis R.A., Dragone G., Mussatto S.I. (2020). Strategies for an improved extraction and separation of lipids and carotenoids from oleaginous yeast. Sep. Purif. Technol..

[30] Martínez J., Schottroff F., Haas K., Fauster T., Sajfrtová M., Álvarez I., Raso J., Jaeger H. (2020). Evaluation of pulsed electric fields technology for the improvement of subsequent carotenoid extraction from dried Rhodotorula glutinis yeast. Food Chem..

[31] Martínez J.M., Delso C., Aguilar D.E., Álvarez I., Raso J. (2020). Organic-solvent-free extraction of carotenoids from yeast Rhodotorula glutinis by application of ultrasound under pressure. Ultrason. Sonochem..

[32] Martínez J.M., Delso C., Angulo J., Álvarez I., Raso J. (2018). Pulsed electric field-assisted extraction of carotenoids from fresh biomass of Rhodotorula glutinis. Innov. Food Sci. Emerg. Technol..

[33] Hernández-Almanza A., Navarro-Macías V., Aguilar O., Aguilar-González M., Aguilar C.N. (2017). Carotenoids extraction from Rhodotorula glutinis cells using various techniques: A comparative study. Indian J. Exp. Biol..

[34] Cardoso L., Jäckel S., Karp S., Framboisier X., Chevalot I., Marc I. (2016). Improvement of Sporobolomyces ruberrimus carotenoids production by the use of raw glycerol. Bioresour. Technol..

[35] Wang H.-B., Zhang L.-W., Luo J., Yu L.-J. (2015). Erratum to: Rapid and environmentally-friendly extraction of carotenoids from Blakeslea trispora. Biotechnol. Lett..

[36] Michelon M., Borba T.D.M.D., Rafael R.D.S., Burkert C.A.V., Burkert J.F.D.M. (2012). Extraction of carotenoids from Phaffia rhodozyma: A comparison between different techniques of cell disruption. Food Sci. Biotechnol..

[37] Hardesty J.H., Attili B. (2010). Spectrophotometry and the Beer-Lambert Law: An important analytical technique in chemistry. Collin Coll. Dep. Chem..

[38] Filho J.L.E.D., Maia P.C.D.A., Xavier G.D.C. (2019). Spectrophotometry as a tool for characterizing durability of woven geotextiles. Geotext. Geomembr..

[39] Lipson R.H. (2009). Ultraviolet and Visible Absorption Spectrometers. Encycl. Appl. Spectrosc..

[40] Feng Y., Shao Y., Chen F. (2012). Monascus pigments. Appl. Microbiol. Biotechnol..

[41] Patakova P. (2013). Monascus secondary metabolites: Production and biological activity. J. Ind. Microbiol. Biotechnol..

[42] Laqua K., Melhuish W.H., Zander M. (1988). Molecular Absorption Spectroscopy, Ultraviolet and Visible (Uv/Vis). Pure Appl. Chem..

[43] Molnár P., Ősz E., Turcsi E., Deli J. (2019). Carotenoid composition of the mushroom Scarlet elf cup (Sarcoscypha coccinea). Heliyon.

[44] Moussa S.A.-K., Abdou D.A., Mohamed G.A., Abo-El-Seoud M.A., Eldin A.-Z.K., El-Mehalawy A.A. (2018). Production of red pigment by Monascus purpureus NRRL1992 under submerged and solid-state fermentation. Egypt. J. Microbiol..

[45] Gunasekaran S.M., Poorniammal R.M. (2008). Optimization of fermentation conditions for red pigment production from Penicillium sp. under submerged cultivation. Afr. J. Biotechnol..

[46] Whetsel K.B. (1968). Near-Infrared Spectrophotometry. Appl. Spectrosc. Rev..

[47] Jennings G., Bluck L., Wright A., Elia M. (1999). The Use of Infrared Spectrophotometry for Measuring Body Water Spaces. Clin. Chem..

[48] Quijano-Ortega N., Fuenmayor C.A., Zuluaga-Dominguez C., Diaz-Moreno C., Ortiz-Grisales S., García-Mahecha M., Grassi S. (2020). FTIR-ATR Spectroscopy Combined with Multivariate Regression Modeling as a Preliminary Approach for Carotenoids Determination in Cucurbita spp.. Appl. Sci..

[49] Gerrard D.L., Bowley H.J. (1989). Instrumentation for Raman Spectroscopy. Practical Raman Spectroscopy.

[50] Adadi P., Barakova N.V., Krivoshapkina E.F. (2018). Selected Methods of Extracting Carotenoids, Characterization, and Health Concerns: A Review. J. Agric. Food Chem..

[51] Movasaghi Z., Rehman S., Rehman I.U. (2007). Raman Spectroscopy of Biological Tissues. Appl. Spectrosc. Rev..

[52] Nokkaew R. (2019). Determination of carotenoids and dobi content in crude palm oil by spectroscopy techniques: Comparison of raman and ft-nir spectroscopy. Int. J. Geomate.

[53] Rajawat J., Jhingan G. (2019). Mass spectroscopy. Data Processing Handbook for Complex Biological Data Sources.

[54] Dole M., Cox H.L., Gieniec J., Ezrin M. (1973). Electrospray Mass Spectroscopy. Polymer Molecular Weight Methods.

[55] Fan-Chiang H.-J., Wrolstad R.E. (2006). Anthocyanin Pigment Composition of Blackberries. J. Food Sci..

[56] Chen M., Zhang T.T., He L., Wang K., Chen Y. (2021). Qualitative analysis of cotton fiber pigment composition. Text. Res. J..

[57] Steyn P.S., Wessels P.L., Marasas W.F. (1979). Pigments from fusarium moniliforme sheldon. Tetrahedron.

[58] Rangasami R., Chidhara S., Chandrasekharan A. (2016). Magnetic resonance imaging and magnetic resonance spectroscopy in Salmonella meningoencephalitis. J. Pediatr. Neurosci..

[59] Shi Q., Wang H., Du C., Zhang W., Qian H. (2013). Tentative Identification of Torulene *Cis/trans* Geometrical Isomers Isolated from *Sporidiobolus pararoseus* by High-Performance Liquid Chromatography—Diode Array Detection—Mass Spectrometry and Preparation by Column Chromatography. Anal. Sci..

[60] Liu C., Cheng Y., Du C., Lv T., Guo Y., Han M., Pi F., Zhang W., Qian H. (2019). Study on the wall-breaking method of carotenoids producing yeast *Sporidiobolus pararoseusand* the antioxidant effect of four carotenoids on SK-HEP-1 cells. Prep. Biochem. Biotechnol..

[61] Kopáni M., Celec P., Danišovič L., Michalka P., Biró C. (2006). Oxidative stress and electron spin resonance. Clin. Chim. Acta.

[62] Barba F.J., Roohinejad S., Ishikawa K., Leong S.Y., Bekhit A.A.E.-D., Saraiva J.A., Lebovka N. (2020). Electron spin resonance as a tool to monitor the influence of novel processing technologies on food properties. Trends Food Sci. Technol..

[63] Passos M.L., Saraiva M.L.M. (2019). Detection in UV-visible spectrophotometry: Detectors, detection systems, and detection strategies. Measurement.

[64] Heikkilä T.T., Silaev M., Virtanen P., Bergeret F.S. (2019). Thermal, electric and spin transport in superconductor/ferromagnetic-insulator structures. Prog. Surf. Sci..

[65] Torgerson D., Skowronski R., Macfarlane R. (1974). New approach to the mass spectroscopy of non-volatile compounds. Biochem. Biophys. Res. Commun..

[66] Gessler N.N., Egorova A.S., Belozerskaya T.A. (2014). Melanin pigments of fungi under extreme environmental conditions (Review). Appl. Biochem. Microbiol..

[67] Gonçalves R.C.R., Lisboa H.C.F., Pombeiro-Sponchiado S.R. (2011). Characterization of melanin pigment produced by *Aspergillus nidulans*. World J. Microbiol. Biotechnol..

[68] Grosser K., Van Dam N.M. (2017). A Straightforward Method for Glucosinolate Extraction and Analysis with High-pressure Liquid Chromatography (HPLC). J. Vis. Exp..

[69] Sahu P.K., Ramisetti N.R., Cecchi T., Swain S., Patro C.S., Panda J. (2018). An overview of experimental designs in HPLC method development and validation. J. Pharm. Biomed. Anal..

[70] Dalal R.C., Henry R.J. (1986). Simultaneous Determination of Moisture, Organic Carbon, and Total Nitrogen by Near Infrared Reflectance Spectrophotometry. Soil Sci. Soc. Am. J..

[71] Tavares D.G., Barbosa B.V.L., Ferreira R.L., Duarte W.F., Cardoso P.G. (2018). Antioxidant activity and phenolic compounds of the extract from pigment-producing fungi isolated from Brazilian caves. Biocatal. Agric. Biotechnol..

[72] Esser N., Speiser E. (2018). Introduction to Raman scattering at surfaces. Physics of Solid Surfaces.

[73] Poole C.F. (2003). Thin-layer chromatography: Challenges and opportunities. J. Chromatogr. A.

[74] Stillwell W. (2016). Membrane Reconstitution. An Introduction to Biological Membranes.

[75] Hostettmann K., Marston A. (1995). Saponins. Saponins.

[76] Bates C.J. (2005). Fat-Soluble. Vitamin D.

[77] Blanc P., Loret M., Santerre A., Pareilleux A., Prome D., Prome J., Laussac J., Goma G. (1994). Pigments of Monascus. J. Food Sci..

